# Cognitive Dysfunctions and Assessments in Multiple Sclerosis

**DOI:** 10.3389/fneur.2019.00581

**Published:** 2019-06-04

**Authors:** Celia Oreja-Guevara, Teresa Ayuso Blanco, Luis Brieva Ruiz, Miguel Ángel Hernández Pérez, Virginia Meca-Lallana, Lluís Ramió-Torrentà

**Affiliations:** ^1^Servicio de Neurología, Hospital Clínico San Carlos, IdISSC, Departamento de Medicina, Universidad Complutense, Madrid, Spain; ^2^Complejo Hospitalario de Navarra, Navarra, Spain; ^3^Hospital Arnau de Vilanova de Lleida, IRBLLEIDA, Lleida, Spain; ^4^Servicio de Neurología, Hospital Universitario Nuestra Señora de Candelaria, Universidad de La Laguna, Santa Cruz de Tenerife, Spain; ^5^Unidad de Esclerosis Múltiple, Servicio de Neurología, Fundación de Investigación Biomédica, Hospital Universitario de la Princesa, Madrid, Spain; ^6^Unidad de Esclerosis Múltiple y Neuroinmunología de Girona, Servicio de Neurología, IDIBGI, Hospital Universitario Dr. Josep Trueta, Girona, Spain

**Keywords:** multiple sclerosis, cognitive impairment, cognitive dysfunction, memory, attention, neuropsychology, processing speed, depression

## Abstract

Cognitive impairment has been reported at all phases and all subtypes of multiple sclerosis. It remains a major cause of neurological disability in young and middle-aged adults suffering from the disease. The severity and type of cognitive impairment varies considerably among individuals and can be observed both in early and in later stages. The areas which have commonly shown more deficits are: information processing speed, complex attention, memory, and executive function. Even though an alteration in both the white matter and in the gray matter has been found in patients with multiple sclerosis and cognitive impairment, the underlying process still remains unknown. Standardized neurological examinations fail to detect emerging cognitive deficits and self-reported cognitive complaints by the patients can be confounded by other subjective symptoms. This review is a comprehensive and short update of the literature on cognitive dysfunctions, the possible confounders and the impact of quality of life in patients with multiple sclerosis.

## Introduction

Multiple sclerosis (MS) is a chronic, inflammatory and autoimmune demyelinating neurodegenerative disease of the central nervous system that can affect different functions and that is clinically characterized by relapses, remissions, and progression of disability over time (1, 2). Jean-Martin Charcot was the first to describe cognitive impairment in multiple sclerosis in the nineteenth century (3). Specifically, cognitive impairment can affect up to 70% of patients with MS (4), it has been reported at all phases and all subtypes of the disease (5); even though it is more frequent in the secondary progressive type (6) and is a major cause of neurological disability in young and middle-aged adults (2). The severity and type of cognitive impairment varies considerably among individuals and can be observed in early and in later stages of the disease (7, 8). The areas which have commonly shown more deficits are: information processing speed, episodic memory, complex attention, and executive function (6, 9). There are cognitive domains such social cognition (10) and moral cognition (11, 12), which have been less explored in patients with MS.

Cognitive decline reduces the work productivity of these patients (13, 14) and can have great effect in the quality of life of the persons with MS (15) which can also be impaired by the effects the disease has in other areas (16, 17).

In patients with MS and cognitive impairment an alteration both in the white matter (WM) and in the gray matter (GM) of the brain has been found (18–20). However, the complete etiology remains unclear, as little is still known about their relative contribution to the underpinning process of cognitive impairment. There is a generally poor correlation between the symptoms of cognitive impairment and the conventional MRI measures of structural damage (21).

Risk factors for the onset and development of cognitive impairment in patients with MS have been described elsewhere (22). These include individual differences between patients, such as age, sex, personality traits and health behavior; genetic factors and MRI alterations in the brain (18). Cognitive reserve is paramount, for it has an impact as a prognostic factor (23). The paradox between the burden of the disease, physical disability and T2 lesions and the cognitive state is largely explained by the cognitive reserve. Patients with MS and higher education, more vocabulary, more hobbies and more activities with high cognitive performance have more cognitive reserve (20, 23).

The assessment of cognitive function in routine clinical practice is still undervalued, despite a lot of published and validated cognitive tests and batteries. It is necessary that clinical neurologists know the need and benefits of performing cognitive assessments in patients with MS routinely (24). Standardized neurological examinations may be unable to detect emerging cognitive deficits and self-reported cognitive complaints by the patients (25), which can also be confounded by other subjective symptoms, such as depression and anxiety (26). It is therefore paramount to include an early screening that detects impaired cognition in patients with MS (27). This could allow for an earlier intervention (28), which would also include a better psychoeducation and specific work with patients with MS and their families (27).

A systematic review was conducted searching using identical search terms across electronic databases: Pubmed, Embase, Web of Science and PsycInfo. The inclusion criteria were articles published in peer-reviewed journals in the last 15 years that included participants aged between 18 to 65 years. Clinical practice guidelines were also reviewed.

The aim of this paper is to review the published literature in a simple and structured way and to write a short and comprehensible updated review about the cognitive dysfunctions, their assessments, the possible confounders, and the impact of quality of life in patients with MS.

## Cognitive Dysfunctions in MS

The pattern of cognitive impairment in MS has been defined as “disconnection syndrome” or “fronto-subcortical syndrome” even though other structures are also implied (29). We review the most affected cognitive domains in patients with MS.

### Information Processing

The information processing speed can be affected in 40–70% of the patients with MS (30). The efficiency in information processing in MS refers both to working memory—to maintain and manipulate information for a short period of time—and to the processing speed—the speed at which a certain series of cognitive operations can be performed. Both are affected in MS and interact with each other, although some authors believe that it is more frequent to find the processing speed affected, especially in patients with a secondary progressive form (30).

The slowing in information processing seems to be the most frequent cognitive alteration in MS and one of the first cognitive symptoms that can be detected (30–32). This can also affect the ability to follow a certain conversation.

Among the tests used to evaluate processing speed are SDMT (Symbol Digit Modalities Test) (evaluates visual processing speed) and PASAT (Paced Auditory Serial Addition Test) (auditory). When comparing the performance between patients with MS and healthy controls, greater effect sizes were evidenced with the SDMT (33). For this reason, the SDMT is considered the measure of choice for MS trials in assessing cognitive processing speed (34). In addition, some authors have tested an auto-SDMT, which is entirely computer administrated, uses speech recognition technology to allow to do in more patients without the need for a human tester (35).

### Memory

Memory difficulties have been found in 40–65% of patients, with 30% of patients having severe memory problems (36).

In MS the alterations occur mainly at the explicit memory (declarative), having to do with deliberate recall and the recovery about personal experiences and the knowledge of the world. Generally, there is preservation of implicit memory (non-declarative), in which previous experiences facilitate the execution of a task, without a conscious perception of it (37).

According to Tulving's traditional classification, this explicit memory would be subdivided into episodic and semantic memory (38). Within the explicit memory, this episodic memory would be the most affected in patients with MS (39). In this way, the complaints in consultation are related to forgetfulness (36). Some studies suggest that the difficulty might lie in patients' inability to retrieve long-term storage information, while others point out that the source of the problem lies in the initial learning difficulties, meaning that patients would need a greater number of repetitions to acquire the information, but both recall and recognition would be normal. In this sense, some studies indicate that, if the patient is given more time to process and encode information results significantly improve (36). Thus, it is frequently observed that there is not much difference between the amount of information that is recovered in “immediate recall” and “delayed recall” and there are no differences in this regard between patients with MS and controls. The greatest difficulty in acquiring new information would be associated with executive dysfunction, sensory deficits, slowing of information processing and greater susceptibility to interference that may exist in patients with MS (9).

Tests that evaluate this domain include both auditory-verbal as the Selective Reminding Test (SRT) and California Verbal Learning Test and for visuo-spatial information the Spatial Recall Test (SPART).

### Attention

Between 20 and 50% of the patients have specific attentional difficulties (36). The most affected components of attention in MS are selective, sustained, alternating and divided attention. In contrast, alert level and focused attention are components not so frequently impaired. People with MS most frequently refer to have difficulties with following a conversation or a television program, to keep doing a task at work, to resume a certain activity after an interruption or, to maintain focus on a particular stimulus when other competing stimuli exists.

Alterations at the attention level have been related to difficulties both in working memory and processing speed. Thus, most of the tests that evaluate attention components also take into consideration processing speed and working memory (40).

### Executive Functions

Executive functions are the skills needed to carry out creative, effective and socially accepted behavior, and include a set of processes, including anticipation, planning, goal-setting, and self-regulation.

Between 15 and 25% of patients would show executive difficulties (36), and between 20 and 25% difficulties in verbal fluency tasks (41), making executive alterations less frequent.

### Language and Intelligence

Most studies show that both language and intelligence are generally preserved in MS. However, some authors have shown a slight decline in the IQ (specifically in the manipulative IQ) vs. the preservation of verbal IQ. When the disease begins in childhood, a greater alteration in language and IQ has been described (39).

Basic verbal skills (expression and understanding) are often preserved, except for occasional difficulties in naming. If there are problems in verbal comprehension these seem to be related more to difficulties in information processing or with working memory. The most prevalent verbal difficulty is the low performance in verbal fluency tasks (especially phonemic fluency over semantic), which are more often related to executive functioning (36), while aphasias are infrequent.

### Visuoperceptive Functions

The main alterations have to do with face recognition and angle matching (36). Although visual disturbances such as optic neuritis may exert a negative influence on perceptual processing, perceptual deficits have been observed regardless of the existence of primary visual impairment in up to 25% of patients (42).

### Social Cognition

Social cognition is the individual's ability to understand own and others minds and feelings in order to give adequate answers in the person's social environment (43). We can also define it as the way we perceive the social world. It is one of the six core functional cognitive domains. MS has been associated with social cognition impairment, which is sometimes overlooked and which might have a drastic impact on the quality of life and the social relationships (10, 44). Deficits in the theory of mind and facial emotion recognition were identified among patients with MS, especially in older patient (45). Social cognition can be evaluated using different tests: Reading the Mind in the Eyes Test, Faces Test, Faux-Pas Test and the MASC (Movie Assessment Social Cognition) examination. It seems that fatigue, an invisible symptom of MS, might have a correlation with social cognition performance, which could be due to common underlying neuronal networks (46). Mindfulness-based intervention (MBI) could be useful to improve social cognition (47).

### Neuropsychological Assessment

There is no agreement on what should be the most suitable instruments for the exploration of cognitive impairment in MS. Batteries of short and large tests are available. Short evaluation instruments select the most sensitive and specific tests to obtain the information in a shorter period of time, minimizing costs and are also very effective in the monitoring of the deficits during follow up. It is very important to carry out a neuropsychological screening evaluation that can identify cognitive impairment, before a more extensive and comprehensive evaluation is performed. The recent consensus of the national MS society for cognitive screening (48) recommends that a minimum early baseline screening should be done and it can be with the SDMT and moreover it is very important to do an annual reassessment with the same test or battery for all adults with MS.

There are different kinds of batteries (Table 1):

**Table 1:** Neuropsychological assessment in patients with MS.

	**BRB-N**	**Portaccio**	**BNB**	**MACFIMS**	**BICAMS**
Administration Time	30 min	< 30 min	< 30 min	90 min	< 90 min
Sensitivity	71%	94%	-	-	94%
Specificity	94%	84%	-	-	84%
Tests	SRT, SPART, SDMT, PASAT, WLG	SRT, SDMT, PASAT-3	Verbal episodic memory test, SDMT, verbal fluency tasks, PASAT	CVLT-II, BVMTR, D-KEFS, JLO, COWAT	CVLT-II, BVMT-R, SDMT

*BRB-N, Brief Repeatable Battery of Neuropsychological Test; Portaccio, Brief battery of Portaccio; BNB, Short Neuropsychological Battery; MACFIMS, Minimal Assessment of Cognitive Function in MS; BICAMS, Brief International Cognitive Assessment for Multiple Sclerosis; SRT, Selective Reminding Test; SPART, 10/36 Spatial Recall Test; SDMT, Symbol Digit Modalities Test; PASAT, Paced Auditory Serial Addition Test; WLG, Word List Generation; CVLT-II, Verbal California Learning Test-II; BVMTR, Visuospatial Memory Test Revised; D-KEFS, The Delis Kaplan Executive Functioning system sorting test; JLO: Judgment of line orientation test; COWAT, Controlled Oral Word Association Test*.

### Brief Repeatable Battery of Neuropsychological Test (BRB-N)

This is the most used neuropsychological battery both in clinical practice and in research (41).

It has the advantages that it is administered in a relatively short period of time (30 min) and that each of the 5 tests of which it is composed has 15 alternative forms, so that the battery can be used longitudinally. It has a high sensitivity (71%) and specificity (94%) for the identification of the explored cognitive domains. It evaluates verbal and visual episodic memory, complex attention, information processing speed, and some aspects of executive function. The included tests are:

- SRT: assesses learning ability and verbal long-term retention. This test distinguishes between short-term and long-term memory, and the difficulty of learning or retrieving information.
- 10/36 SPART: assesses learning capacity and long-term visuospatial retention.
- SDMT: evaluates the sustained attention and the capacity of concentration, besides the visuomotor speed. Differences have been observed in the performance of this test between healthy controls and patients with MS (49). It has proven to be the most sensitive test to detect cognitive impairment over time (50).
- PASAT: evaluates working memory and sustained attention.
- Word List Generation (WLG): evaluates semantic verbal fluency through categorial evocation.

### Brief Battery of Portaccio

It is a short version of BRB-N that includes SRT, SDMT and PASAT-3. This battery is able to detect cognitive impairment with a sensitivity of 94%, a specificity of 84%, and a precision of 89% (51).

### Short Neuropsychological Battery (BNB)

This is a Spanish proposal (31) which includes a verbal episodic memory test, SDMT, several verbal fluency tasks and an adapted version of the PASAT test in which the patient is allowed more time. Visual-spatial memory is not evaluated. It requires a shorter time of application than the BRB-N. BNB is a rapid screening test to assess the cognitive impairment of patients with MS. The failure in at least two tests (≥ 1.5 Standard Deviations) is considered as a presence of cognitive impairment.

### Minimal Assessment of Cognitive Function in MS (MACFIMS)

The MACFIMS battery is actually an extension of the classic BRB-N battery (52). It has a considerable consensus and a high reliability test/retest. The Visuospatial Memory Test Revised (BVMTR) and the Verbal California Learning Test-II (CVLT-II) are also used. The Delis Kaplan Executive Functioning system sorting test (D-KEFS) is also added to evaluate executive function and the JLO-Judgment of line orientation test to evaluate spatial function and Controlled Oral Word Association Test (COWAT) to assess verbal fluency. This battery is completed in approximately 90 min.

### Brief International Cognitive Assessment for Multiple Sclerosis (BICAMS)

It is a reduced version of the MACFIMS (53), which includes the California Verbal Learning Test-II (CVLT II), in addition to SDMT and Brief Visuospatial Memory Test Revised (BVMT-R). It is an international and easy scale to perform. The sensitivity obtained with this battery is 94% with a specificity of 84% (54). This battery has been validated in different languages. Moreover, cut points are trying to be established to allow clinicians to identify patients with possible cognitive impairment to guide clinical decision-making. Beier et al. (55) proposed that cut scores can accurately identify cognitive impairment on all subtests of the BICAMS.

## Frequent Confounders of Cognition

When assessing cognitive impairment, several confounders must be taken into account. Among them, anxiety and depression stand as the most important. Other frequent confounders are fatigue and sleep disorders. Furthermore, some disease-modifying treatments (DMTs) have depression as a side effect and some symptomatic treatments for spasticity or pain may induce sedation or dizziness, which both have a great impact on the cognitive status (56).

### Depression

Depression in MS affects approximately 37–54% of patients (57), being the most frequently diagnosed psychiatric disorder in the disease, with an estimated risk of occurrence of 50%, compared to 10–15% in the normal population (58–60).

Its etiology is usually considered multifactorial. Depression is also more frequent in MS than in other chronic diseases that in a similar way cause physical or cognitive disability, so there must be other associated causes to explain their occurrence. Although results from several studies have shown that depression is associated with neuropsychological functioning in MS patients, other investigations have not found this association (61–66). Different studies have shown that depressive disorders are more frequent in patients with cognitive impairment. The association of cognitive dysfunction and depression leads to worse results in different neuropsychological tests than those without a depressive symptomatology (66).

Depression in MS may involve different neuropsycho-biological mechanisms (67) and it is postulated that the existence (or not) of coping strategies could be a crucial factor. An explanatory model has been proposed, taking into account variables such as social support, coping strategies, stress, self-knowledge and perception of the disease, all of which would influence modulating the onset of depression (68). Patients affected with MS and cognitive impairment and with major depressive symptomatology use few coping strategies and, on the contrary, employ a high degree of negative coping or avoidance strategies.

Depression affects many aspects of cognitive function in MS, which include working memory, information processing speed, learning functions, abstract reasoning, and executive functioning (69–73), so improvement of depression could improve the functioning of patients in these aspects (69).

The depressive symptomatology is closely related to the reduction of the patient's quality of life (59), causes greater absenteeism (74) and reduces adherence to treatment (73).

Depression in MS may be related to the treatments being used; in particular, immunomodulatory drugs such as interferon beta, although some studies do not find this association (73, 75, 76). For the evaluation of depression, the most commonly used classification is diagnostic and statistical manual for mental disorders (DSM-5) (77), and there are different scales for assessing depression in MS patients (78): Beck Depression Inventory (79), Hospital Anxiety and Depression Scale (80), Patient Health Questionnaire (PHQ-9) (81), Center for Epidemiologic Studies Depression Rating Scale (CESD) (82),Chicago Multi-Scale Depression Inventory (CMDI)(83), Hamilton Rating Scale for Depression (84), Depressive Mood Scale (85), and Zung Self Rating Depression Scale (ZSRD) (86) (Table 2).

**Table 2:** Scales assessing depression in MS patients.

	**BDI**	**HADS**	**PHQ-9**	**CES-D**
Self-administered	Yes	Yes	No	Yes
Assesses	Clinical symptoms of melancholia Intrusive thoughts	Anxiety Depression	Depressive symptoms present in the last 2 weeks	Depressive symptoms present in the last week
Cut-off points	0-13: minimum depression 14-19: mild depression 20-28: moderate depression 29-63: severe depression	The score range is 0-21 for each subscale, and 0-42 for the overall score. A score of ≥ 11 in a subscale suggests that there might be a clinical problem	The range of scores is 0-27 and each item ranges from 0 (never) to 3 (more than half the days)	Major depressive episode: anhedonia or dysphoria nearly every day for the past 2 weeks, plus symptoms in an additional 4 DSM symptom groups noted as occurring nearly every day for the past 2 weeks
Advantages	Has the highest percentage of cognitive symptoms present, emphasizing the absence of motor symptoms and anxiety	Suppression of somatic symptoms: depression can be evaluated independently of the underlying somatic disease	A short version has been proposed using two screening questions for the detection of major depressive disorders in the context of MS and primary care (PHQ-2) corresponding to depressed mood (“during the last 2 weeks, have you felt down, depressed, or hopeless?“) and anhedonia (“have you felt little interest or pleasure in doing things for the past 2 weeks?“)	Very brief scale, no trained staff is needed
Validated in MS?	No	Yes	Yes	Yes
		Scores on the depression subscale ≥ 8 have shown a sensitivity of 85% and a specificity of 82.2%	Scores ≥ 10 have shown a sensitivity of 95% and a specificity of 85.9%	Scores ≥ 16 have shown a sensitivity of 94.7% and a specificity of 73.1%

*BDI, Beck Depression Inventory; HADS, Hospital Anxiety and Depression Scale; PHQ-9, Patient Health Questionnaire, CES-D, Center for Epidemiologic Studies Depression Rating Scale*.

Both psychopharmacological treatment (antidepressants) and psychotherapeutic interventions like mindfulness should be used for depressive symptoms, as they have shown benefits in the management of depression and anxiety (87).

### Anxiety

Compared with depression, anxiety is a poorly studied symptom in MS with few studies evaluating the relationship between anxiety and cognitive performance (78). Its prevalence is estimated to be between 12 and 40% (88). It has been associated with the presence of fatigue, pain, degree of disability, rates of suicidal ideation, and little adherence to treatment (78, 89, 90).

Anxiety affected significantly MS patients from controls (91). Higher anxiety rates have also been reported in newly diagnosed patients (34%) (92).

The most commonly used instrument to assess anxiety is the HADS anxiety subscale (84).

### Fatigue and Sleep Disorders

Fatigue is one of the most common symptoms of MS and it is one of the so-called invisible symptoms, which are very difficult to measure in an objective way. Fatigue in MS affects approximately 70–90% of patients (13, 17). It usually limits daily functional activities, which has a negative impact on the quality of life.

Similar to what has been described for cognition, it is assumed that fatigue is a result of network disruption which includes frontal, parietal, temporal and occipital regions, thalami, and basal ganglia (93, 94). Processing speed, assessed with the SDMT is lower in patients reporting fatigue (95).

Moreover, fatigue is a stronger predictor of self-reported cognitive function 2 years later than depression (96).

Patients with sleeping problems also have more fatigue and more cognitive dysfunctions. It seems that 19–67% of people with MS suffer from a sleep disorder (97). Obstructive sleep apnea and sleep disorders are associated with decreased visual and verbal memory, executive function, attention and processing speed (48, 97, 98).

## Impact of Cognition in Quality of Life

World Health Organization (WHO) defines quality of life as individuals' perception of their position in life in the context of the culture and value systems in which they live and in relation to their goals, expectations, standards and concerns (99). It is a broad concept that encompasses physical, psychic and social factors and is concerned with whether disease or impairment limits a person's ability to fulfill a normal role (100).

Quality of life is not as well-evaluated as in other chronic diseases (101, 102), mainly because: MS has multiple clinical manifestations, it affects mainly young adults (20–40 years) in which they will have to readjust their life expectations and the prognosis of MS is uncertain.

The studies which take into consideration health in relation with quality of life have made their own contributions. Identification of disease impact areas that had not been considered by conventional clinical scales (103–105), they reveal important perceptual differences between doctors and their patients: as patients show more concern for aspects such as fatigue, sexuality or vitality than to their physical disability, it allows identifying and assessing both the patient's needs and the existence of possible complications not yet detected by the physician (102) and they are able to predict and anticipate the clinical course of the disease (99, 100, 106).

Cognitive impairment increases patient morbidity and is associated with a decrease in the participation and functioning of activities of daily living (103), such as driving, medical decision-making and treatment adherence, money management, and work (48). Cognitive impairment appears to be associated with increased rates of unemployment and lower measures of QOL (107). The most common scales to measure quality of life in cognitive studies are EuroQOL five dimension questionnaire, a generic health-related QOL scale and the Short Form 36 (SF-36) questionnaire (107, 108).

## Discussion

Cognitive impairment is a common, yet challenging expression of MS (106). It is a frequent cause of disability and socioeconomic decline for patients with MS (13, 105). Even though the capability of detecting cognitive difficulties has increased over the last years, there are still many patients who remain undiagnosed and whose complaints are considered to be part of comorbidities. Besides, the options to treat these symptoms are still scarce. Sometimes treating the depression, anxiety and fatigue by psychotherapeutic interventions improves the cognitive alterations (87).

No direct relationship has been yet found between cognitive impairment and the clinical course of the disease (109). Thus, cognitive deficits which occur during the early stages of the disease are the ones which need to be identified and addressed, in order to prevent cognitive impairment from becoming worse, implicating a poor prognosis in MS.

The cognitive domains which show greater levels of impairment have been broadly studied in patients with MS (52). As cognitive function can be reliably assessed in patients with MS, this should be another clinical sign that physicians should bear in mind in order to consider clinical decisions regarding disease-modifying treatment (22). The SDMT test has proved to be a valuable screening tool for cognitive impairment and could be the starting line when assessing cognitive impairment in patients with MS when more comprehensive screening tools or staff resources are not available (27, 50). An annual reassessment with the same test or battery for all adults with MS is strongly recommended (48).

The neuropsychological assessment should also discriminate between cognitive impairment and other causes for perceived deficits, including anxiety, depression and quality of life. As aforementioned, depression in MS seems to involve multiple factors that include neurobiological variables, physical disability, cognitive dysfunction, certain treatments, the uncertainty of the disease itself and the deterioration of the quality of life itself that can contribute as a causal agent but also as a consequence.

## Author Contributions

All authors listed have made a substantial, direct and intellectual contribution to the work, and approved it for publication.

### Conflict of Interest Statement

CO-G has received honoraria for speaking and/or consultancy from Biogen, Genzyme, Bayer, Merck, Roche, Teva, and Novartis. LR-T has received compensation for consulting services and speaking honoraria from Biogen, Novartis, Bayer, Merck, Sanofi, Genzyme, Teva Pharmaceutical Industries Ltd, Almirall, Mylan. VM-L has received consulting or speaking fees from Almirall, Biogen, Genzyme, Merck Serono, Novartis, Roche, Terumo, Sanofi, and Teva. The remaining authors declare that the research was conducted in the absence of any commercial or financial relationships that could be construed as a potential conflict of interest.
